# Sharing Programming Resources Between Bio* Projects

**DOI:** 10.1007/978-1-4939-9074-0_25

**Published:** 2019-01-01

**Authors:** Raoul J. P. Bonnal, Andrew Yates, Naohisa Goto, Laurent Gautier, Scooter Willis, Christopher Fields, Toshiaki Katayama, Pjotr Prins

**Keywords:** Bioinformatics, R, Python, Ruby, Perl, Java, Web services, RPC, EMBOSS, PAML

## Abstract

Open-source software encourages computer programmers to reuse software components written by others. In evolutionary bioinformatics, open-source software comes in a broad range of programming languages, including C/C++, Perl, Python, Ruby, Java, and R. To avoid writing the same functionality multiple times for different languages, it is possible to share components by bridging computer languages and Bio* projects, such as BioPerl, Biopython, BioRuby, BioJava, and R/Bioconductor.

In this chapter, we compare the three principal approaches for sharing software between different programming languages: by remote procedure call (RPC), by sharing a local “call stack,” and by calling program to programs. RPC provides a language-independent protocol over a network interface; examples are SOAP and Rserve. The local call stack provides a between-language mapping, not over the network interface but directly in computer memory; examples are R bindings, RPy, and languages sharing the Java virtual machine stack. This functionality provides strategies for sharing of software between Bio* projects, which can be exploited more often.

Here, we present cross-language examples for sequence translation and measure throughput of the different options. We compare calling into R through native R, RSOAP, Rserve, and RPy interfaces, with the performance of native BioPerl, Biopython, BioJava, and BioRuby implementations and with call stack bindings to BioJava and the European Molecular Biology Open Software Suite (EMBOSS).

In general, call stack approaches outperform native Bio* implementations, and these, in turn, outperform “RPC”-based approaches. To test and compare strategies, we provide a downloadable Docker container with all examples, tools, and libraries included.

## 1 Introduction

Bioinformatics has created its tower of Babel. The full set of functionality for bioinformatics, including statistical and computational methods for evolutionary biology, is implemented in a wide range of computer languages, e.g., Java, C/C++, Perl, Python, Ruby, and R. This comes as no surprise, as computer language design is the result of multiple trade-offs, for example, in strictness, convenience, and performance. In this chapter we discuss strategies for combining solutions from different languages and look at performance implications of combining cross-language functionality. In the process we also highlight implications of such strategic choices.

Computer languages used in bioinformatics today typically fall into two groups: those compiled and those interpreted. Java, C++, and D, for example, are statically typed compiled languages, while R, Perl, Ruby, and Python are dynamically typed interpreted languages. In principle, a compiled language is converted into machine code once by a language compiler, and an interpreted language is compiled every time at runtime, the moment it is run by an interpreter. Static typing allows a compiler to optimize machine code for speed. Dynamic typing requires an interpreter and resolves variable and function types at runtime. Such design decisions cause Java, C++, and D to have stronger compile-time type checking and faster execution speed than R, Perl, Ruby, and Python. When comparing runtime performance of these languages, compiled statically typed languages, such as C++, D, and Java, generally outperform interpreted dynamically typed languages, such as Python, Perl, and R. For speed comparison between languages, see, for example, the benchmarks game.

Statically typed compiled languages tend to produce faster code at runtime

Runtime performance, however, is not the only criterion for selecting a computer language. R, Perl, Ruby, and Python offer sophisticated interactive analysis of data in an interpreted shell which is not directly possible with C++, D, or Java. Another important criterium may be conciseness. Interpreted languages generally allow functionality to be written in less lines of code. The number of lines matter, as it is often easier to grasp something expressed in a short and concise fashion, if done competently, leading to easier coding and maintenance of software and resulting in increased programmer productivity. In general, with R, Perl, Ruby, and Python, it takes less lines of code to write software than with C++, D, or Java; this is also visible from the examples in the benchmarks game.

Interpreted languages allow for concise code that is easier to read and results in increased programmer productivity

Based on the conciseness criterium, computer languages fall into these two groups. This suggests a trade-off between execution speed and conciseness/programmer productivity. Even so, strong typing may help later when refactoring code, perhaps regaining some of that lost productivity. The authors also note that in their experience, the more programming languages one masters, the easier it becomes mastering new languages (with the exception, perhaps, of Haskell). Learning new programming languages is important when writing software.

Logically, to fully utilize the potential of existing and future bioinformatics functionality, it is necessary to bridge between computer languages. Bioinformaticians cannot be expected to master every language, and it is inefficient to write the same functionality for every language. For example, R/Bioconductor contains unique and exhaustive functionalities for statistical methods, such as for gene expression analysis [1]. The singular implementation of this functionality in R has caused researchers to invest in learning the R language. Others, meanwhile, have worked on building bridges between languages. For example, RPy and Rserve allow accessing R functionality from Python [2], and JRI and Rserve allow accessing R functionality from Java [3, 4]. Other languages have similar bindings, such as RSRuby that allows accessing R from Ruby.

Discussing other important criteria for selecting a programming language, such as ease of understanding, productivity, portability, and the size and dynamics of the supporting Bio* project developer communities, is beyond the scope of this chapter. The authors, who have different individual preferences, wish to emphasize that every language has characteristics driven by language design and there is no single perfect all-purpose computer language. In practice, the choice of a computer language depends mainly on the individuals involved in a project, partly due to the investment it takes to master a language. Researchers and programmers have prior investments and personal preferences, which have resulted in a wide range of computer languages used in the bioinformatics community.

Contrasting with singular implementations, every mainstream Bio* project, such as BioPerl [5], Biopython [6], BioRuby [7], R/Bioconductor [1], BioJava [8], the European Molecular Biology Open Software Suite (EMBOSS) [9], and Bio++ [10], contains duplication of functionality. Every Bio* project consists of a group of volunteers collaborating at providing functionality for bioinformatics, genomics, and life science research under an open-source software (OSS) license. The BioPerl project does that for Perl, BioJava for Java, etc. Next to the language used, the total coverage of functionality, and perhaps quality of implementation, differs between projects. Not only is there duplication of effort, both in writing and testing code, but also there are differences in implementation, completeness, correctness, and performance. For example, implementations between projects differ even for something as straightforward as codon translation, e.g., in number of types of encoding and support for the translating of ambiguous nucleotides. EMBOSS, uniquely, attempts to predict the final amino acid in a sequence, even when there are only two nucleotides available for the last codon.

Whereas Chapter 25 discusses Internet data resources and how to share them, in this chapter, we discuss how to share functional resources by interfacing and bridging functionality between different computer languages. This is highly relevant to evolutionary biology as most classic phylogenetic resources were written in C, while nowadays phylogenetic routines are written in Java, Perl, Python, Ruby, and R. Especially for communities with relatively few software developers, we argue here that it is important to bridge these functional resources from multiple languages. For bridging, strategies are here discussed to invoke one program from another, use some form of remote procedure calls (RPC), or use a local call stack.

### 1.1 Bridging Functional Resources Calling from Program to Program

The most simple way of interfacing software is by invoking one program from another. This strategy is often used in Bio* projects, for example, for invoking external programs. A regular subset would be PAML [11], HMMER [12], ClustalW [13], MAFFT [14], Muscle [15], BLAST [16], and MrBayes [17]. The Bio* projects typically contain modules which invoke the external program and parse the results. The advantage of this approach is that it mimics running a program on the command line, so invocation is straightforward. Another advantage, in a web service context, is that if the called program crashes, it does not have to take the whole service down. There are also some downsides, however. Loading a new instance of a program every time incurs extra overhead. More importantly, nonstandard input and output makes the interface fragile, i.e., what happens when input or output differs between two versions of a program? A further downside is that external programs do not have fine-grained function access and have no support for advanced error handling and exceptions. What happens, for example, when the invoked program runs out of process memory? How to handle that gracefully? A final complication is that such a program is an external software deployment dependency, which may be hard to resolve for an end user.

### 1.2 Remote Procedure Call

In contrast to calling one program from another, true cross-language interfacing allows one language to access functions and/or objects in another language, as if they are native function calls. To achieve transparent function calls between different computer languages, there are two principal approaches. The first approach is for one language to call directly into another language’s function or method over a network interface, the so-called remote procedure call (RPC). The second approach is to call into another language over a local “call stack.”

In bioinformatics, cross-language RPC comes in the form of web services and binary network protocols. A web service application programming interface (API) is exposed, and a function call gets translated with its parameters into a language-independent format, a procedure called “marshalling.” After calling the function on a server, the result is returned in, for example, XML and translated back through “unmarshalling.” Examples of cross-language XML protocols are SOAP [18] and XML/RPC [19].

More techniques exist for web service-type cross-language RPC. For example, representational state transfer (REST), or ReSTful [20], is a straightforward HTTP protocol, often preferred over SOAP because of its simplicity. Another XML-based protocol is Resource Description Framework (RDF), as part of the semantic web specification. Both REST and RDF can be used for RPC solutions.

In addition, binary alternatives exist because XML-based protocols are not very efficient. XML is verbose, increasing the data load, and requires parsing at both marshalling and unmarshalling steps. In contrast, binary protocols are designed to reduce the data transfer load and increase speed. Examples of binary protocols are Rserve [3], which is specifically designed for R, and Google protocol buffers [21]. Another software framework based on a binary protocol is Thrift, by the Apache software foundation, designed for scalable cross-language service development [22]. Finally, also worth considering are very fast interoperable messaging-based paradigms, such as ZeroMQ [23], and high-level message-level optimizers, such as GraphQL.

### 1.3 Local Call Stack

The alternative to RPC is to create native local bindings from one language to another using a shared native call stack, essentially linking into code of a different computer language. With the call stack, function calls do not run over the network but over a stack implementation in shared computer memory. In a single virtual machine, such as the JVM and Erlang Beam, compiled code can share the same call stack, which can make cross-language calling efficient. For example, the languages Java, Jython, JRuby, Clojure, Groovy, and Scala can transparently call into each other when running on the same virtual machine using native speeds.

Native call stack sharing is also supported at the lowest level by the computer operating system through compiled shared libraries. These shared libraries have an extension .so on Linux, .dylib on OSX, and .dll on Windows. The shared libraries are designed so that they contain code and data that provide services to independent programs, which allows the sharing and changing of code and data in a modular fashion. Shared library interfaces are well defined at the operating system level, and languages have a way of binding them. Specialized interface bindings to shared libraries exist for every language, for example, R’s C modules, the Java Native Interface (JNI) for the JVM, Foreign Function Interfaces (FFI) for Python and Ruby, the Parrot native compiler interface PerlXS for Perl.

With (dynamic) shared libraries, certain algorithms can be written in a low-level, high-performance compiled computer language, such as C/C++, D, or FORTRAN. And high-level languages, such as Perl, Python, Ruby, R, and even Java, can access these algorithms. This way, languages can be mixed to optimize solutions. Creating these shared library interfaces, however, can be a tedious exercise, which often calls for code generators. One such generator is the Simplified Wrapper and Interface Generator (SWIG) [24], which consists of a macro-language, a C header file parser, and the tools to bind low-level shared libraries to a wide range of languages. For C/C++, SWIG can parse the header files and generate the bindings for other languages, which, in turn, call into these shared libraries. The Boost project has similar facilities for mapping calls to SWIG. C FFI’s that come with programming languages, such as Python’s CFFI and Ruby’s FFI, tend to be the easiest to work with.

Even though this extensive functionality for interfacing is available, the full potential of creating cross-language adapters is not fully exploited in bioinformatics. Rather than bridge two languages, researchers often opt to duplicate functionality. This is possibly due to a lack of information on the effort involved and the added complexity of creating a language bridge. Also, the impact on performance may be an unknown quantity. A further complication is the need to understand, to some degree, both sides of the equation, i.e., to provide an R function to Python requires some understanding of both R and Python, at least to the level of reading the documentation of the shared module and creating a working binding. Likewise, binding Python to C using a call stack approach requires some understanding of both Python and C. Sometimes, binding of complex functions can be daunting, and deployment may be a concern, e.g., when creating shared library bindings on Linux, they may not easily work on Windows or macOS.

### 1.4 Comparing Approaches

Here, we compare bridging code from one language to another using the RPC approach and the call stack approach. As a comparison we also provide a program-to-program approach and show how dependencies can be fixated. The comparison is done in the form of short experiments (scripts) which can be executed by the reader. To measure performance between different approaches, we use codon translation as an example of shared functionality between Bio* projects. Codon translation is a straightforward algorithm with table lookups. Such sequence translation is representative of many bioinformatics tasks that deal with genome-sized data and require many function calls with small-sized parameters.

In this chapter we first focus on comparing R and Python bindings. We include native Bio* implementations, i.e., Biopython, BioRuby, BioPerl, BioJava, and EMBOSS (C) for an absolute speed comparison. Next we try bindings on the JVM.

Examples and tests can in principle be experimented with a computer running Linux, macOS, or Windows. To ease trials, we have defined GNU Guix packages that contain the tools and their dependencies. From this we have created a downloadable Docker image that supports all interfaces and performance examples (GNU Guix and Docker are discussed in Chapter 25).

## 2 Results

### 2.1 Calling into R

R is a free and open-source environment for statistical computing and graphics [25]. R comes with a wide range of functionality, including modules for bioinformatics, such as bundled in R/Bio-conductor [1]. R is treated as a special citizen in this chapter because the language is widely used and comes with statistical algorithms for evolutionary biology, such as Ape [26] and SeqinR [27], both available through the comprehensive R archive network (CRAN).

R defines a clear interface between the high-level language R and low-level highly optimized C and FORTRAN libraries, some of which have been around for a long time, such as the libraries for linear regression and linear algebra. In addition, the R environment successfully handles cross-platform packaging of C, C++, FORTRAN, and R code. The combination of features has resulted in R becoming the open-source language of choice in a number of communities, including statistics and some disciplines in biology. R/Bioconductor has gene expression analysis [1] and R/qtl [28] and R/qtlbim [29] for QTL mapping (see also QTL mapping in Chapter 21). Not all is lost, however, for those not comfortable with the R language itself. R can act as an intermediate between functionality and high-level languages. A number of libraries have been created that interface to R from other languages, either providing a form of RPC, through RSOAP or Rserve, or a call stack interface calling into the R-shared library and executing R commands, for example, RPy for Python, RSPerl for Perl, RSRuby for Ruby, and JRI for Java. Of the last call stack approaches, RPy currently has the most complete implementation; see also [2].

**Fig. 1:**
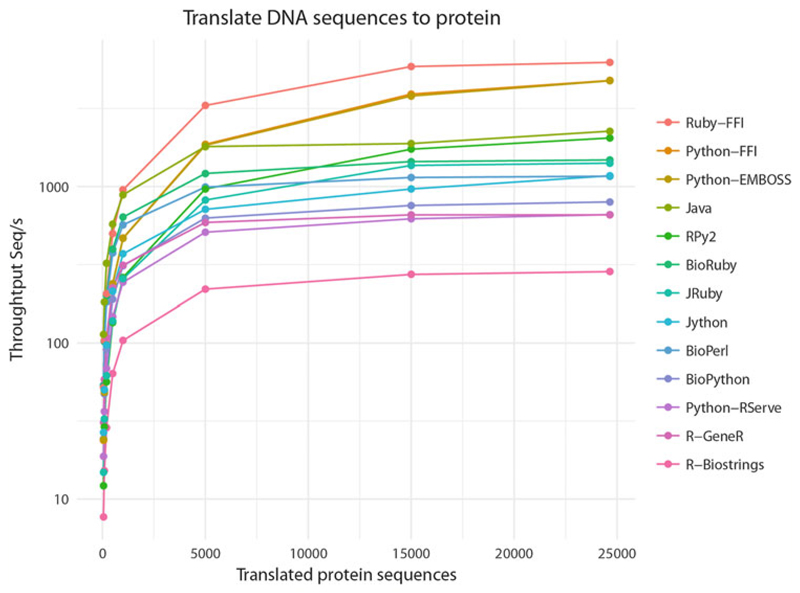
Throughput of mRNA to protein translation using combinations of cross-language calling with a range of programming resources. WormBase *C. elegans* predicted protein coding DNA that was parsed in FASTA format and translated into amino acids. Tests were executed inside a container. Different file sizes were used containing 500, 1000, 5000, 15,000, and 25,000 sequences (*X*-axis) and the number of sequences processed per seconds (*Y*-axis log_10_ scale). Measurements were taken on an AMD Opteron(TM) 6128 8 cores at 2.0 GHz, 4 sockets x 8 cores, with 512 GB RAM DDR3 ECC, and an HDD SATA of 2 TB. Broadly the figure shows that sustained throughput is reached quickly and flattens out. R-Biostrings performs poorly at 285 Seq/s, while R-GeneR and Rserve (Python+Rserve+GeneR) perform at the level of native Bio* libraries, respectively, 658 Seq/s and 660 Seq/s. The cross-language Ruby-FFI at 6256 Seq/s calls EMBOSS C translation and outperforms all others

In this chapter, we compare different approaches for invoking full R functionality from another language. To test cross-language calling, we elected to demonstrate codon translation. Codon-to-protein amino acid translation is representative for a relatively simple computation that potentially happens thousands of times with genome-sized data. Every Bio* project includes such a translation function, so it is a fair way to test for language interoperability and performance. For data, we use a WormBase [30] C. *elegans* cDNA FASTA file (33 Mb), containing 24,652 nucleotide sequences, predicted to translate to protein (Fig. 1).

#### 2.1.1 Using GeneR with Plain R

“The R/Bioconductor GeneR package [31] supports fast codon translation with the strTranslate function implemented in C.” GeneR supports the eukaryotic code and other major encoding standards. R usage is: library(GeneR)
strTranslate("atgtcaatggtaagaaatgtatcaaatcagagcgaaaaattggaaattttgt")
[1] "MSMVRNVSNQSEKLEIL"

The \name{R+GeneR} script (also available here) reads: fasta = ’dna.fa’
library(GeneR)
idx = indexFasta(fasta)
lines <-readLines( paste(fasta,’.ix’,sep=’’) )
index <-read.table(paste(fasta,’.ix’,sep=’’) )[,1]
n = 0
for (i in 1:times) {
  for (name in index) {
   readFasta (file=fasta, name = name)
   ntseq = getSeq(0)
   aaseq = strTranslate(ntseq)
   cat(">",name," (",n,")\n",aaseq,"\n",sep="")
   n = n+1
  }
}

and parses the nucleotide FASTA input and outputs amino acid FASTA. Run the script: docker run --rm -v ‘pwd‘/tmp:/tmp -v ‘pwd‘/scripts:/scripts -e \
  BATCH_VARS=/tmp/test-dna-${i}.fa -t bionode bash -c "source
/etc/profile
cd /book-evolutionary-genomics
./scripts/create_test_files.rb
R -q --no-save --no-restore --no-readline --slave < src/R/
DNAtranslate_GeneR.R" > /dev/null

Used directly from R, the throughput of the GeneR module is about 658 sequences per second (Seq/s) on the test system, an AMD Opteron(TM) 6128 CPU at 2.00 GHz (*see* also Fig. 1). When checking the implementation by reading the source code, in the first edition, we found that the GeneR FASTA parser was a huge bottleneck. The FASTA parser implementation created an index on disk and reloaded the full index file from disk for each individual sequence, thereby incurring a large overhead for every single sequence.

To see if we could improve throughput, we replaced the slow FASTA parser with \name{R+Biostrings} which reads FASTA once into RAM using the R/Bioconductor BioStrings module and still uses GeneR to translate. At the time, this implementation was 1.6 times faster than GeneR. At this time GeneR is 3.2 on average faster than reading with Biostrings which had a throughput of 284.83 Seqs/s proving some work was done by the authors to improve GeneR. The second script can be found here.

#### 2.1.2 Calling into R from Other Languages with RPC

One strategy for bridging between languages is to use R as a network server and invoke remote procedure calls (RPC) over the network.

1. SOAP
  SOAP allows processes to communicate using XML over HTTP in a client/server setup. SOAP is an operating system and computer language “agnostic,” so it can be used to bridge between languages. In the previous edition of this chapter {Ref to Previous Edition, same chapter}, we wrote a R/SOAP [32] adapter for codon translation and invoked it from Python (a Python to R bridge). That client script can be found here. The SOAP bridge was dropped from this chapter because the SOAP packages are not maintained and it was by far the slowest method of cross-language interfacing we tried! The marshalling and unmarshalling of simple string objects using XML over a local network interface takes a lot of computational resources. We do not recommend using SOAP.
2. Rserve
  Rserve [3] is a custom binary network protocol, more efficient than XML-based protocols [3]. R data types are converted into Rserve binary data types. Rserve was originally written for Java, but nowadays connectors exist for other languages. With Rserve, Python and R do not have to run on the same server. Furthermore, all data structures will automatically be converted from native R to native Python and numpy types and back.

With RServe fired up a Python example is: import pyRserve
conn = pyRserve.connect()
conn.eval(’library(GeneR)’)
conn.eval(’strTranslate("atgtcaatggtaagaaatgtatcaaatcagagcgaaaaattggaaattttgt")’)
’MSMVRNVSNQSEKLEIL’

where Rserve+GeneR uses the GeneR translate function. In our test Biopython [6] is used for parsing FASTA, and at 797 Seq/s, even with this network bridge, Python+Rserve’s speed is on par with that of R. The script can be found here.

#### 2.1.3 Calling into R from Other Languages with the Call Stack Approach

Another strategy for bridging language is to use a native call stack, i.e., data does not get transferred over the network. RPy2 executes R code from within Python over a local call stack [2]. Invoking the same GeneR functions from Python: import rpy2.robjects as robjects
from rpy2.robjects.packages import importr
importr(’GeneR’)
strTranslate=robjects.r[’strTranslate’]
strTranslate("atgtcaatggtaagaaatgtatcaaatcagagcgaaaaattggaaattttgt") [0]
’MSMVRNVSNQSEKLEIL’

This example uses Biopython for parsing FASTA and invokes GeneR translation over a call stack handled by RPy2. At 2049 Seq/s, throughput is the highest of our calling into R examples. The Python implementation outperforms the other FASTA parsers, and GeneR is fast too when only the translation function is called (GeneR’s strTranslate is actually written in C, not in R). Still, there are some overheads for bridging and transforming string objects from Python into R and back. The RPy2 call stack approach is efficient for passing data back and forth. The script can be found here.

### 2.2 Native Bio* Implementations

When dealing with cross-language transport comparisons, it is interesting to compare results with native language implementations. For example, Biopython [6] would be: from Bio.Seq import Seq
from Bio.Alphabet import generic_dna
coding_dna = Seq("atgtcaatggtaagaaatgtatcaaatcagagcgaaaaattggaaattttgt", generic_dna)
coding_dna.translate()
Seq(’MSMVRNVSNQSEKLEIL’, ExtendedIUPACProtein())

which runs at 797 Seq/s which is slower than the Python3+RPy2+GeneR version. This is because the translate function is written in Python and not in C. It is, however, still faster than R+GeneR. Ruby+BioRuby runs faster at 1481 Seq/s. Perl+BioPerl is in the middle with 1165 Seq/s. We can assume the Biopython, BioPerl, and BioRuby implementations are reasonably optimized for performance. Therefore, throughput reflects the performance of these interpreted languages (*see* Fig. 1).

Java is a statically typed compiled language. Java+BioJava [8] outperforms the interpreters and runs at 2266 Seq/s.

The source code for all examples can be found here in the {Biopython}, {BioRuby}, {BioPerl}, and {BioJava} subdirectories.

### 2.3 Using the JVM for Cross-Language Support

The Java virtual machine (JVM) is a “bytecode” standard that represents a form of computer intermediate language. This language conceptually represents the instruction set of a stack-oriented capability architecture. This intermediate language, or “bytecode,” is not tied to Java specifically, and in the last 10 years, a number of languages have appeared which target the JVM, including JRuby (Ruby on the JVM), Jython (Python on the JVM), Groovy [33], Clojure [34], and Scala [35]. These languages also compile into bytecode and share the same JVM stack. The shared JVM stack allows transparent function calling between different languages.

An example of calling BioJava translation from a Scala program: import org.biojava.nbio.core.sequence.transcription.TranscriptionEngine
import org.biojava.nbio.core.sequence._

val transcriber = TranscriptionEngine.getDefault()
val dna = new DNASequence("atgtcaatggtaagaaatgtatcaaatcagagcgaaaaattggaaattttgt")
val rna = dna.getRNASequence(transcriber)
rna.getProteinSequence(transcriber)
’MSMVRNVSNQSEKLEIL’

which uses the BioJava libraries.

A native Java function, such as getProteinSequence, is directly invoked from the other language without overheads (the passed-in transcriber object is passed by reference, just like in Java). In fact, Scala compiles to bytecode, which maps one to one to Java, including the class definitions. The produced bytecode is a native Java bytecode; therefore, the performance of calling BioJava from Scala or Java is exactly the same. This also holds for other languages on the JVM, such as Clojure and Groovy.

We have also included a JRuby example that calls into BioJava4 on the JVM and runs at 1413 Seq/s. JRuby is an interpreter on the JVM that still needs some translation calling into JVM functions. It is therefore slower than native calls.

### 2.4 Shared C Library Cross-Calling Using EMBOSS Codon Translation

EMBOSS is a free and OSS analysis package specially developed for the needs of the molecular biology user community, mostly written in C [9].

#### 2.4.1 FFI

Using Foreign Function Interface (FFI), it is possible to load dynamic libraries at runtime, define classes to map composite data types, and bind functions for a later use inside your host programming language. We used FFI to bind the EMBOSS translation function to Python and Ruby. The Python example: from ctypes import *
import os
emboss = cdll.LoadLibrary(os.path.join(os.path.dirname(os.
path.abspath(__file__)),"emboss.so"))
trnTable = emboss.ajTrnNewI(1)
ajpseq = emboss.ajSeqNewNameC(b"atgtcaatggtaagaaatgtatcaaatcagagcgaaaaattggaaattttgt",
b"Test sequence")
ajpseqt = emboss.ajTrnSeqOrig(trnTable,ajpseq,1)
seq = emboss.ajSeqGetSeqCopyC(ajpseqt)
seq = str(c_char_p(seq).value,’utf-8’)
print(seq)
MSMVRNVSNQSEKLEILX

The Ruby example: require ’ffi’

module Emboss
  extend FFI::Library
  ffi_lib "./emboss.so"
  attach_function :ajTrnNewI, [:int], :pointer
   attach_function :ajSeqNewNameC, [:pointer, :pointer], :
pointer
  attach_function :ajTrnSeqOrig, [:pointer, :pointer, :int], :
pointer
  attach_function :ajSeqGetSeqCopyC, [:pointer], :string
end

trnTable = Emboss.ajTrnNewI(1)
ajpseq = Emboss.ajSeqNewNameC("atgtcaatggtaagaaatgtatcaaatcagagcgaaaaattggaaattttgt",
"Test sequence")
ajpseqt = Emboss.ajTrnSeqOrig(trnTable,ajpseq,1)
aa = Emboss.ajSeqGetSeqCopyC(ajpseqt)
print aa,"\n"
MSMVRNVSNQSEKLEILX

In both cases the advantage of FFI is that it does not require to compile any source code, just loading the shared library and binding what is needed. Python has a native library called ctypes, and more sophisticated libraries are available to help the programmer bind complex data structures and functions. Ruby has a dedicated gem called [ruby-ffi].

**Fig. 2:**
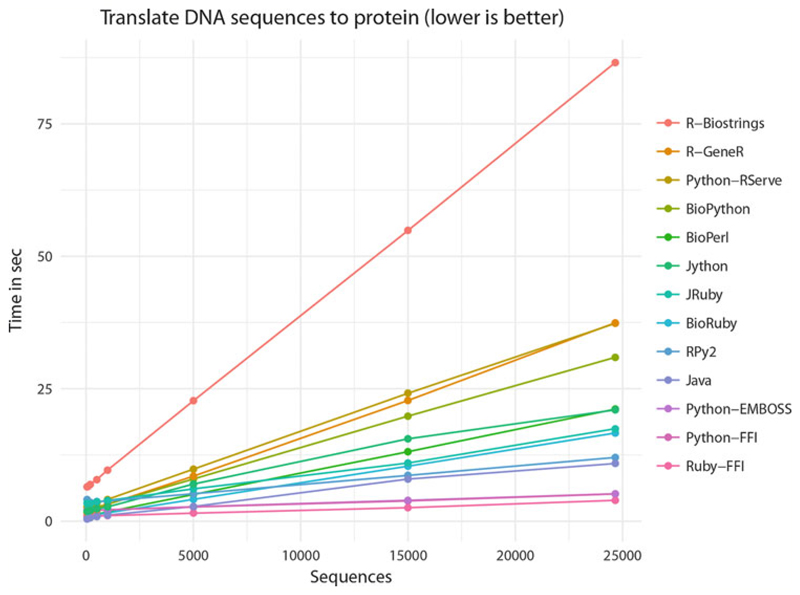
Number of seconds needed for processing mRNA to protein translation using cross-language calling with a range of programming resources. *See* Fig. 1 for the setup. The figure shows that for all the implementations, the time increases linearly with the number of sequences in input. R-Biostrings performs poorly with an upstart of 6.50 s and the highest slope. The cross-language Ruby-FFI, Python FFI, and Python-EMBOSS with an upstart slightly higher than Java have a very minimal slope; Ruby-FFI has a nearly constant time

The Ruby and Python FFI outperforms all above methods at 6257 Seq/s and 4787 Seq/s, respectively (*see* Fig. 1). Plotting the time in seconds spent to translate the sequences, Ruby and Python FFI are the lowest (quickest) in the whole comparison (*see* Fig. 2). The high speed points out that (1) the invoked Biopython and BioRuby functions are reasonably efficient at parsing FASTA, (2) the FFI-generated call stack is efficient for moving data over the local call stack, and (3) the EMBOSS transeq DNA to protein translation is optimal C code.

### 2.5 Calling Program to Program

Calling program to program is far more common than you may think because even when you run a program in a shell, such as Bash, you are calling program to program. You can invoke EMBOSS from the command line: transeq test-dna.fa test.pep

transeq is written in C and runs at a very fast 23,478 Seq/s. Invoking above EMBOSS’ transeq in Python looks like this: os.system("transeq "+fn+" out.pep")
for seq_record in SeqIO.parse("out.pep", "fasta"):
    print(">",seq_record.id)
    seq = str(seq_record.seq)
    print(seq)

and this combination runs at 4768 Seq/s. That is close to Python FFI and a third of the speed of transeq on its own because of Python parsing the output. Every parsing step has a cost attached.

### 2.6 Web Services

A discussion on bridging languages would not be complete if we did not include web services, particularly using REST API’s. Service like TogoWS and EBI web services which include EMBOSS transeq (SOAP) offer functionality over http(s) and can be used from any programming language. Here a Ruby example of using TogoWS: ## Invoke irb by loading BioRuby
% irb -r bio

## Create a TogoWS object
>> togows = Bio::TogoWS::REST.new
=>  #<Bio::TogoWS::REST:0x007f840faab9d8 @pathbase="/",
@http=#<Net::HTTP togows.dbcls.jp:80 open=false>,
  @header={"User-Agent"=>"BioRuby/1.5.1"}, @debug=false>

## Search for UniProt entries by keywords
>> togows.search(’uniprot’, ’lung cancer’)
=> "KKLC1_MACFA\nKKLC1_HUMAN\nDLEC1_HUMAN\n .....

## Retrieve one UniProt entry (or multiple entries if you like)
>> entry = togows.entry(’uniprot’, ’KKLC1_MACFA’)

## See the entry content
>> puts entry
ID  KKLC1_MACFA             Reviewed;        114 AA.
AC  Q4R717;
 :
## Convert the retrieved UniProt entry into FASTA format
>> puts togows.convert(entry, ’uniprot’, ’fasta’)
>KKLC1_MACFA RecName: Full=Kita-kyushu lung cancer antigen
1 homolog;
MNVYLLLASGILCALMTVFWKYRRFQRNTGEMSSNSTALALVRPSSTGLINSNTDNNLSV
YDLSRDILNNFPHSIAMQKRILVNLTTVENKLVELEHILVSKGFRSASAHRKST

Web services can harness a lot of power because they use large databases and access up-to-date information. As an example, let’s generate RDF from above entry: ## Retrieve PubMed entry and convert it into RDF/Turtle
(or JSON or XML if you like)
>> puts togows.entry(’pubmed’, ’16381885’, ’ttl’)
@prefix dc: <http://purl.org/dc/elements/1.1/> .
@prefix dcterms: <http://purl.org/dc/terms/> .
@prefix rdfs: <http://www.w3.org/2000/01/rdf-schema#> .
@prefix prism: <http://prismstandard.org/namespaces/2.0/basic/> .
@prefix medline: <http://purl.jp/bio/10/pubmed/> .

<http://rdf.ncbi.nlm.nih.gov/pubmed/16381885>   medline:pmid
"16381885" ;
        rdfs:label      "pmid:16381885" ;
        dc:identifier   "16381885" ;
        medline:own     "NLM" ;

Unfortunately, data centric web services can be slow, i.e., sending and retrieving data over the internet incurs large latency and throughput penalties. Sometimes they use powerful back ends, and it is possible to submit large batch jobs which compete with locally installed solutions. Examples are the BLAST service [16] and GeneNetwork [36].

## 3 Discussion

The half-life of bioinformatics software is 2 years—Pjotr Prins

In this chapter we show that there are many ways of bridging between computer languages. Cross-language interfacing is a topic of importance to evolutionary genomics (and beyond) because computational biologists need to provide tools that are capable of complex analysis and cope with the amount of biological data generated by the latest technologies. Cross-language interfacing allows sharing of code. This means computer software can be written in the computer language of choice for a particular purpose. Flexibility in choice of computer programming language allows optimizing of computational resources and, perhaps even more important, software developer resources, in bioinformatics.

When some functionality is needed that exists in a different computer language than the one used for a project, a developer has the following options: either rewrite the code in the preferred language, essentially a duplication of effort, or bridge from one language to the other. For bridging, there are essentially two technical methods that allow full programmatic access to functionality: through RPC or a local call stack. A third option may be available when functionality can be reached through the command line, as shown above with transeq.

RPC function invocation, over a network interface, has the advantage of being language agnostic and even machine independent. A function can run on a different machine or even over the Internet, which is the basis of web services and may be attractive even for running services locally. RPC XML-based technologies, however, are slow because of expensive parsing and high data load. Our metrics suggest that it may be worth experimenting with binary protocols, such as Rserve and Apache Thrift.

When performance is critical, e.g., when much data needs to be processed, or functions are invoked millions of times, a native call stack approach may be preferred over RPC. Metrics suggest that the EMBOSS C implementation performs well and that binding to the native C libraries with FFI is efficient (*see* Fig. 2). Alternatively, it is possible to use R as an intermediate to C libraries. Interestingly, calling R libraries, many of which are written in C, may give higher performance than calling into native Bio* implementations. For example, Python+RPy2+GeneR is faster that Biopython pure Python implementation of sequence translation, and it is also faster than R calling into GeneR directly—confirming a common complaint that R can be slow.

Even though RPC may perform less well than local stack-based approaches, RPC has some real advantages. For example, if you have a choice of calling a local BLAST library or call into a remote and ready NCBI RPC interface, the latter lacks the deployment complexity. Also the public resource may be more up to date than a copied server running locally. This holds for many curated services that involve large databases, such as PDB [37], Pfam [38], KEGG [39], and UniProt [40]. Chapter 25 gives a deeper treatment of these Internet resources.

From the examples given in this chapter, it may be clear that actual invocation of functions through the different technologies is similar, i.e., all listed Python scripts look similar, provided the underlying dependencies on tools and libraries have been resolved. The main difference between implementations is with deployment of software, rather than invocation of functionality. The JVM approach is of interest, because it makes bridging between supported languages transparent and deployment straightforward. Not only can languages be mixed, but also the advanced Java tool chain is available, including debuggers, profilers, load distributors, and build tools. Other shared virtual machines, such as .NET and Parrot, potentially offer similar advantages but are less used in bioinformatics.

In the first edition, we wrote that when striving for reliable and correct software solutions, the alternative strategy of calling computer programs as external units via the command line should be discouraged: not only is it less efficient that a program gets started every time a function gets called, but also a potential deployment nightmare is introduced. What happens when the program is not installed, or the interface changed between versions, or when there is some other error? With the full programmatic interfaces, discussed in this chapter, incompatibilities between functions get caught much earlier. In this edition of the chapter, we add that efficiency considerations still hold, and error handling can be problematic. When it comes to deployment, however, there now exist solutions that fixate versions of software and give control of the dependency graph, i.e., a tool like transeq can be coupled with its exact version against your software. To ascertain coupling: first there are containers, such as offered by Docker, that allow for bundling software binaries. Second, some recent software distributions allow for formal deployment solutions with reproducible dependency graphs. If you want to know more, check the GNU Guix and NixOS projects. It is possible to combine these deployment technologies. In fact, with this chapter, we provide tools and scripts defined as GNU Guix packages and hosted in a Docker container. These solutions are discussed in Chapter 25.

Choosing a computer language should not be based on runtime performance considerations alone. The maturity of the language and accompanying libraries, tools, and documentation should count heavily, as well as the activity of the community involved. The time saved by using a known language versus learning a new language should be factored in. The main point we are trying to make here is that it is possible to mix languages using different interfacing strategies. This allows leveraging existing functionality, as written by others, using a language of choice. Depending on one’s needs, it is advisable to test possible alternatives for performance, as the different tests show that performance varies.

Whichever language and bridging technology is preferred, we think it important to test the performance of different ways of interfacing languages, as there is (1) a need for combining languages in bioinformatics and (2) it is not always clear what impact a choice of cross-language interface may have on performance. By testing different bridging technologies and functional implementations, the best solution should emerge for a specific scenario.

So far, we have focused on the performance of cross-language calling. In Chapter 25, scalability of computation is discussed by programming for multiple processors and machines.

## 4 Questions

1. Install the Docker container and run different tests. Can you replicate the differences of throughput statistics?
2. Why are network protocols such as Rserve slower than native call stack approaches?
3. What are possible advantages of using a virtual machine, such as the JVM?
4. If you were to bridge between your favorite language and an R library, what options do you have?

## References

[1] Gentleman RC, Carey VJ, Bates DM (2004). Bioconductor: open software development for computational biology and bioinformatics. Genome Biol.

[2] Gautier L (2010). An intuitive Python interface for Bioconductor libraries demonstrates the utility of language translators. BMC Bioinformatics.

[3] Urbanek S (2003). Rserve a fast way to provide R functionality to applications. Proceedings of the 3rd International Workshop on Distributed Statistical Computing (DSC 2003).

[4] Urbanek S (2009). How to talk to strangers: ways to leverage connectivity between R, Java and objective C. Comput Stat.

[5] Stajich JE, Block D, Boulez K (2002). The Bioperl toolkit: Perl modules for the life sciences. Genome Res.

[6] Cock PJ, Antao T, Chang JT (2009). Biopython: freely available Python tools for computational molecular biology and bioinformatics. Bioinformatics.

[7] Goto N, Prins P, Nakao M (2010). Bioruby: bioinformatics software for the Ruby programming language. Bioinformatics.

[8] Holland RC, Down TA, Pocock M (2008). BioJava: an open-source framework for bioinformatics. Bioinformatics.

[9] Rice P, Longden I, Bleasby A (2000). EMBOSS: the european molecular biology open software suite. Trends Genet.

[10] Dutheil J, Gaillard S, Bazin E (2006). Bio++: a set of C++ libraries for sequence analysis, phylogenetics, molecular evolution and population genetics. BMC Bioinformatics.

[11] Yang Z (1997). PAML: a program package for phylogenetic analysis by maximum likelihood. Comput Appl Biosci.

[12] Eddy SR (2008). A probabilistic model of local sequence alignment that simplifies statistical significance estimation. PLoS Comput Biol.

[13] Larkin MA, Blackshields G, Brown NP (2007). Clustal W and clustal X version 2.0. Bioinformatics.

[14] Katoh K, Kuma K, Toh H, Miyata T (2005). MAFFT version 5: improvement in accuracy of multiple sequence alignment. Nucleic Acids Res.

[15] Edgar RC (2004). MUSCLE: a multiple sequence alignment method with reduced time and space complexity. BMC Bioinformatics.

[16] Altschul SF, Madden TL, Schaffer AA (1997). Gapped BLAST and PSI-BLAST: a new generation of protein database search programs. Nucleic Acids Res.

[17] Ronquist F, Huelsenbeck JP (2003). MrBayes 3: Bayesian phylogenetic inference under mixed models. Bioinformatics.

[18] Box D, Ehnebuske D, Kakivaya G (2000). Simple object access protocol (SOAP) 1.1.

[19] St Laurent S, Johnston J, Dumbill E (2001). Programming Web services with XML-RPC.

[20] Richardson L, Ruby S (2007). Restful web services.

[21] Muller J, Lorenz M, Geller F, Zeier A, Plattner H (2010). Assessment of communication protocols in the EPC network-replacing textual SOAP and XML with binary google protocol buffers encoding.

[22] Agarwal A, Slee M, Kwiatkowski M (2007). Thrift: scalable cross-language services implementation.

[23] Hintjens P (2013). Zeromq: messaging for many applications.

[24] Beazley D (1996). SWIG: an easy to use tool for integrating scripting languages with C and C++.

[25] Development Core Team R (2010). R: a language and environment for statistical computing.

[26] Paradis E, Claude J, Strimmer  K (2004). APE: analyses of phylogenetics and evolution in R language. Bioinformatics.

[27] Charif D, Thioulouse J, Lobry JR, Perriere G (2005). Online synonymous codon usage analyses with the ade4 and seqinR packages. Bioinformatics.

[28] Arends D, Prins P, Jansen RC, Broman KW (2010). R/qtl: high-throughput multiple QTL mapping. Bioinformatics.

[29] Yandell BS, Mehta T, Banerjee S (2007). R/qtlbim: QTL with Bayesian interval mapping in experimental crosses. Bioinformatics.

[30] Harris TW, Antoshechkin I, Bieri T (2010). WormBase: a comprehensive resource for nematode research. Nucleic Acids Res.

[31] Cottret L, Lucas A, Marrakchi E GeneR: R for genes and sequences analysis.

[32] Warnes G (2004). RSOAP provides a SOAP interface for the open-source statistical package R.

[33] Koenig D, Glover A, King P, Laforge G, Skeet J (2007). Groovy in action.

[34] Halloway S (2009). Programming Clojure.

[35] Odersky M, Altherr P, Cremet V (2004). An overview of the Scala programming language. LAMP-EPFL (IC/2004/64).

[36] Sloan Z, Arends D, Broman KW (2016). Genenetwork: framework for web-based genetics. J Open Source Soft.

[37] Berman HM, Battistuz T, Bhat TN (2002). The protein data bank. Acta Crystallogr D Biol Crystallogr.

[38] Finn RD, Mistry J, Tate J (2010). The Pfam protein families database. Nucleic Acids Res.

[39] Kanehisa M, Goto S (2000). KEGG: kyoto encyclopedia of genes and genomes. Nucleic Acids Res.

[40] Bairoch A, Apweiler R, Wu CH (2005). The universal protein resource (UniProt). Nucleic Acids Res.

